# Group foraging increases foraging efficiency in a piscivorous diver, the African penguin

**DOI:** 10.1098/rsos.170918

**Published:** 2017-09-27

**Authors:** Alistair M. McInnes, Cuan McGeorge, Samuel Ginsberg, Lorien Pichegru, Pierre A. Pistorius

**Affiliations:** 1 DST/NRF Centre of Excellence at the Percy FitzPatrick Institute, Department of Zoology, Nelson Mandela Metropolitan University, Summerstrand 6031, South Africa; 2 CapeNature, Stony Point, Betty's Bay 7141, South Africa; 3 Department of Electrical Engineering, University of Cape Town, Rondebosch 7701, South Africa

**Keywords:** foraging theory, hunting strategies, seabirds, Benguela

## Abstract

Marine piscivores have evolved a variety of morphological and behavioural adaptations, including group foraging, to optimize foraging efficiency when targeting shoaling fish. For penguins that are known to associate at sea and feed on these prey resources, there is nonetheless a lack of empirical evidence to support improved foraging efficiency when foraging with conspecifics. We examined the hunting strategies and foraging performance of breeding African penguins equipped with animal-borne video recorders. Individuals pursued both solitary as well as schooling pelagic fish, and demonstrated independent as well as group foraging behaviour. The most profitable foraging involved herding of fish schools upwards during the ascent phase of a dive where most catches constituted depolarized fish. Catch-per-unit-effort was significantly improved when targeting fish schools as opposed to single fish, especially when foraging in groups. In contrast to more generalist penguin species, African penguins appear to have evolved specialist hunting strategies closely linked to their primary reliance on schooling pelagic fish. The specialist nature of the observed hunting strategies further limits the survival potential of this species if Allee effects reduce group size-related foraging efficiency. This is likely to be exacerbated by diminishing fish stocks due to resource competition and environmental change.

## 1. Introduction

The ephemeral and evasive nature of shoaling pelagic fish imparts a challenge to marine predators capitalizing on this food source. Marine piscivores have evolved a diversity of morphological and behavioural adaptations to optimize their foraging efficiency. These include the development of functional colorations that facilitate the capture of schooling prey, such as countershading and bold lateral markings in certain cetacean and seabird species [1–3] and the deployment of bubbles by Humpback Whales *Megaptera novaeangliae* to contain schooling prey for subsequent consumption, so-called ‘bubble-netting’ [4]. Group foraging enhances the foraging performance of many marine piscivores either indirectly through facilitation, e.g. sailfish *Istiophorus platypterus* [5], or through well-coordinated cooperative strategies as has been documented for certain Delphinid species [6,7].

Group foraging is commonplace among many seabird species with benefits of foraging flocks including improved location of prey patches through local enhancement [8], and an increase in *per capita* catch rates due to the collective disruption of fish schools [9,10]. For penguins (Spheniscidae), that pursue their prey at depth, there is no empirical evidence for benefits of group foraging despite the knowledge that many penguin species associate among conspecifics at sea and dive synchronously [11–13]. Reliance on group foraging tactics may also be a critical limiting factor for threatened species at a population level as Allee effects [14] can result from diminished foraging efficiency associated with decreasing population densities [15].

The African penguin *Spheniscus demersus*, a Benguela bioregion endemic, has undergone a dramatic decrease in its population since the turn of the century which is believed to be largely influenced by a reduction in the availability of their prey [16]. They feed predominantly on small pelagic fish species [16,17] and, although they can dive deeper than 100 m [18], this prey is typically targeted at mean dive depths between 17 and 33 m [19,20]. African penguins are known to associate in groups at sea, synchronize diving activity and perform corralling behaviour around polarized fish schools [21–23]. However, little is known on the range of foraging strategies employed by this species and, as is the case for all penguins, there is no information on the potential benefits associated with group foraging. A potential Allee effect associated with reduced foraging success in smaller populations could be instrumental in driving the decline in numbers of African penguins [22], but this needs confirmation through *in situ* observations of foraging efficiency under various foraging scenarios.

This study examines the hunting strategies of breeding African penguins from Stony Point in the southern Cape, South Africa, which supported 2533 breeding pairs during 2015 (South African Department of Environmental Affairs 2016, unpublished data). Animal-borne video cameras were deployed on adult birds attending chicks to firstly describe their hunting strategies, and secondly, to establish if group foraging conveyed any benefits in terms of foraging efficiency when compared to solitary foraging.

## 2. Methods

### 2.1. Equipment and field procedures

All fieldwork carried out on African penguins was done under permission from the South African Department of Environmental Affairs (permit nos RES 2015/38 and RES 2016/100) and Cape Nature (permit no. AAA007-00209-0056). Animal-borne video recorders (AVR) were deployed on breeding African penguins attending small chicks at Stony Point, South Africa (34°22′22^″^ S, 18°53′42^″^ E), between 2015 and 2016. The AVRs included the following customizations to Replay XD 720 action cameras (http://www.replayxd.com): construction of pressure-proof marine grade aluminium casings and the inclusion of timer-switches. The latter included an 8-bit microcontroller with two connected switches for setting the recording delays and twelve light emitting diodes to indicate the periods selected. The microcontroller was connected to the power and recording buttons on the camera and the software programmed into the microcontroller was designed to stop and start the cameras after the pre-set delays. The AVRs were tube-shaped, and together with the casing weighed 100 g with dimensions 104 × 26 × 28 mm (length × proximal diameter × distal diameter) giving a cross-sectional area of 616 mm^2^ proximally and 530 mm^2^ distally. Devices were attached to the lower backs of the penguins with strips of waterproof Tesa^®^ tape (Beiersdorf AG, Hamburg, Germany) during the evening preceding an anticipated foraging trip. Morphometric measurements included culmen and bill depth to estimate the sex of the birds using a discriminant function analysis [20]. Two measurements of mass were taken: one during deployment and the other when the bird returned to the colony, either on the same day that the bird was at sea and after the bird had time to provision its chicks, between 16.00 and 20.00, or the following morning if the bird could not be located the previous day. AVRs were programmed to divide the battery life into two recording bins of *ca* 35 min each, at sunset and midday to reflect potential temporal differences in diving behaviour [24].

### 2.2. Quantifying penguin behaviour and prey types

Behavioural information was quantified by analysis of the raw footage using VLC media player (VideoLAN, France). Diving events were classified into commuting, foraging and searching dives. Commuting dives were classified as a succession of more than three shallow dives that were distinctly directional, i.e. very little meandering movements, within approximately 5 m of the surface where the water surface was visible in the video frame. Searching and foraging dives were either shallow (less than 5 m) or deep (greater than 5 m), and included visual confirmation of prey capture for foraging dives. Surface behaviour was classified as either resting or preening and for all behaviours the number of conspecifics was recorded.

The following dive phases were inferred for deep dives: descent phase—from the start of a dive throughout the period of decreasing light; bottom phase—typically dark but with constant light levels, and: ascent phase—increasing light up to the surface. Within each of these dive phases the incidence of undulations and rotational movements of the birds were recorded. Undulations were defined as periods of distinct alternating light and dark phases in rapid succession (less than 3 s) in contrast to the prevailing light conditions in a particular dive phase. This behaviour is associated with prey consumption in Magellanic penguins *Spheniscus magellanicus* [25]. Rotational movements were confirmed in relation to non-rotating distant objects, e.g. fish, particulate matter or conspecifics, and were included as an indication of corralling or herding behaviour. The behaviour of penguins at the bottom phase of deep dives was often obscured due to insufficient illumination and there was the possibility that some prey capture events were missed during these phases. However, these events were likely to be infrequent as observed prey captures in deep waters involved undulating movements where light levels were periodically improved.

Prey types were coarsely classed as either single fish or fish schools, the latter defined as ‘synchronized or polarized swimming groups’ [26]. Based on distinct prey pursuit sequences, fish school encounters were further classified into elevated school and bait-ball events. Elevated school events included schools that were pursued from depth by African penguins toward the sea surface and bait-ball events included highly polarized schools that were suspended near the surface; multiple foraging dives into the same school were recognized as a single feeding event. Dive depths were estimated from the decent phase duration using the descent dive rate, 1.22 m s^–1^ [27].

For all foraging dives involving fish schools, each fish caught was classified based on its location relative to the school: escapee—fish disaggregated from the school; school edge—fish caught less than approximately 2 fish lengths from the school edge, and; school centre—fish caught greater than approximately 2 fish lengths from the school edge. To estimate the number of fish in a school, hereafter termed school size, we used software ImageJ (ver. 1.47, http://imagej.nih.gov/ij) to demarcate polygons around the perimeter of each school and calculated the area of an ellipse fitted to this polygon to account for portions of the school that were not in the image frame. We then sub-sampled five rectangles (length = 0.1 × horizontal azimuth of ellipse, width = 0.1 × vertical azimuth) placed in a cross-formation through the schools, averaged the number of fish counted for all sub-samples and extrapolated this average to the projected area of the school.

### 2.3. Influence of prey aggregation and foraging mode on foraging efficiency

To assess potential benefits of group foraging in African penguins, we modelled the interaction of prey type (i.e. single versus school) and foraging mode (i.e. solitary versus group foraging) against catch-per-unit-effort (CPUE) as the dependent variable. CPUE was calculated as the ratio of prey items caught to the time spent diving during a foraging event and was log transformed to approximate a normal distribution. The duration of foraging events that included the pursuit of fish schools incorporated the dive time between the start of the first foraging dive within which the school was encountered to the end of the last foraging dive where pursuit of the same school was terminated, so as to account for multiple foraging dives into the same school. Incomplete events at the start and end of all recording bins were discarded from this analysis. We used a linear mixed effects model (LMM) with bird ID set as a random effect to account for individual variation in foraging efficiency. A continuous time autocorrelation structure of order 1 (corCAR1) with bird ID set as the grouping factor was fitted to the model to account for potential violation of independence. All computations were carried out using software R [28] and using the package ‘nlme’ [29] for fitting the LMM.

Strong concordance between the distribution of penguins and their prey [30] may lead to a positive correlation between the aggregative potential of penguins and local prey abundance. This may bias assessments of group foraging benefits as the incidence of groups, and therefore group foraging, may be contingent on relative prey abundance. We investigated this potential source of bias on the outcomes of the foraging efficiency models by implementing Spearman's rank correlation tests between relative fish abundance estimates (RFA) for each recording bin and the corresponding number of penguin conspecifics (NCON) present during these periods. Two metrics each for NCON and RFA were calculated for each recording bin: NCON—the maximum number of conspecifics encountered (NCON_max_) and the proportion of all dives with conspecific associations (NCON_prop_); RFA was calculated as the estimated number of fish in the largest school encountered (RFA_max_) and the proportion of non-commuting dives with fish school encounters (RFA_prop_). Only recording bins where schools were recorded were used in these assessments.

## 3. Results

Footage from 12 AVRs deployed at Stony Point between August 2015 and October 2016 was retrieved from seven female and five male African penguins. Devices weighed between 2.9 and 4.1% of the weight of adults deployed on, and most birds (10 of the 12) increased their body masses during the period between deployment and when the AVRs were retrieved (mean ± s.d. mass gain: 126 ± 180 g). Two female penguins lost weight (20 and 25 g respectively) the instruments for both of which were retrieved the subsequent day after they were at sea.

### 3.1. Penguin behaviour and prey types

**Figure 1.**
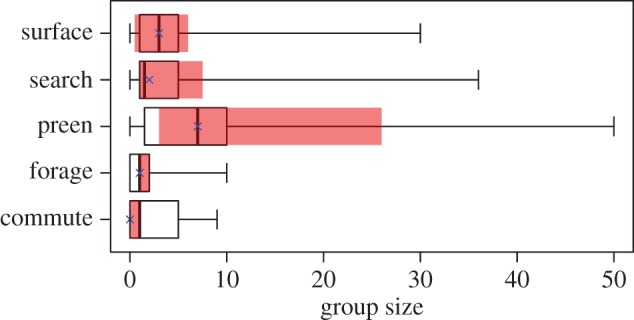
Group sizes for different African penguin behaviours at sea, as observed from animal-borne video recorders. Observations are for the maximum number of conspecifics observed per recording bin showing medians and inter-quartile ranges (IQR) for all individuals (median = vertical bold line, IQR = black box) and only for individuals that participated in group foraging (median = blue cross, IQR = shaded red box).Whiskers represent the ranges for all individuals.

AVR deployments resulted in 820 min of footage (mean ± s.d. time per penguin: 68.3 ± 24.1 min, table 1). A substantial proportion of at-sea activity involved searching and foraging dives (41%) with almost double the time spent searching (27.5%) compared to foraging (13.6%) (table 1). More than a third (34.5%) of all the footage included associations with conspecifics, with preening and foraging constituting the highest (79%) and lowest (13%) incidences of group behaviours, respectively (table 1). African penguins associated in larger groups while preening, especially birds that participated in group foraging (up to 50 birds), compared to other at-sea behaviours with the smallest group sizes recorded for foraging behaviour (figure 1). Foraging activity was recorded in 11 out of the 12 birds (mean ± s.d. total catch per penguin: 31 ± 33) with all prey constituting small pelagic fish, mostly anchovy *Engraulis encrasicolus* (48%) and to a lesser extent juvenile beaked sandfish *Gonorynchus gonorynchus* (3%). A substantial proportion (49%) of fish prey could not be identified to species level.

**Table 1.** At-sea activity budgets of 12 African penguins breeding at Stony Point equipped with animal-borne video recorders (AVRs), showing total duration of each activity (all) and duration only for associations with conspecifics (assoc.).

					surface		commute		preen		search dives		forage dives	
		time			dur. (min)		dur. (min)		dur. (min)		dur. (min)		dur. (min)	
ID	date	start	end	total dur. (m)	all	assoc.	all	assoc.	all	assoc.	all	assoc.	all	assoc.
SP1	20150807	09.45	10.11	26.2	4.1	1.3	2.8	2.2	0.2	0.2	17.4	6.5	1.8	0
SP2	20150820	06.00	11.12	73.1	12	0	30.0	2.4	0	0	15.8	0.3	15.4	0.2
SP3	20150820	06.00	10.54	54.2	29.8	1.1	0	0	5.4	0	18.2	3.9	1.0	0
SP4	20150827	06.45	11.47	61.7	17.1	8.9	7.1	0	0	0	30.7	8.4	6.9	1.7
SP5	20160801	07.30	12.35	64.7	18.5	4.5	10	4.3	13.6	5.9	9.1	3.2	13.6	2.3
SP6	20160802	07.30	12.27	57.6	0.3	0.3	43.5	42.8	12.3	12.3	1.6	1.6	0	0
SP7	20160812	07.30	13.35	64.7	4.1	1.2	49	9.4	0	0	4.1	0.3	7.6	0.6
SP8	20160819	07.30	13.11	101.2	20.5	4.3	20.5	2.5	6.3	6.3	50.6	6.9	3.3	0
SP9	20160929	07.00	13.34	94.2	17.4	10.6	0	0	48.1	48.1	15.5	1.5	13.2	2.9
SP10	20160930	06.15	11.43	89.1	28.9	14.5	0	0	12.7	12.7	29.5	11.8	18.0	1.9
SP11	20161005	06.30	14.38	98.5	30.5	13.3	9.8	0	12.4	0.8	17.7	1.6	28.1	3.9
SP12	20161014	12.10	12.44	35	9.2	2	1	0	6.3	6.3	15.3	4.4	3.2	1.1
totals:				820.1	192.1	61.8	173.5	63.7	117.2	92.5	225.5	50.3	111.9	14.5
% assoc.				34.5		32.2		36.7		79		22.3		12.9

Foraging dives involving pursuit of single fish prey were approximately twice as numerous as dives involving fish schools. The majority (68%) of single prey pursuit dives were deep dives (mean ± s.d.: 37 ± 17 m) despite similar average catch rates between shallow and deep dives (table 2). Dive durations when encountering schooling prey were on average half as long for dives with conspecifics compared to solitary dives, largely due to greater variation in the duration of shallow group dives (table 2). Foraging movements involving undulations were common for most dive types for both single and schooling prey but rotational movements were only recorded for penguins pursuing schooling fish and were most frequent during the ascent phase of dives with conspecifics; these dives were also the most profitable in terms of catches (table 2).

**Table 2.** Behavioural attribute and catch summary for African penguins targeting different prey assemblages (single fish prey and fish schools) during different dive types (all—all dive types combined; shallow—dives less than 5 m, deep—dives greater than 5 m) and dive phases in the presence (association—ass.) and absence (solitary—sol.) of conspecifics. For schooling prey, catch locations are relative to the fish school: escapees—disaggregated from school; school edge—fish less than 2 fish lengths of the school perimeter; school centre—fish greater than 2 fish lengths of the school perimeter. Movement behaviours, undulations and rotations, are given as the proportion of these incidents per event type. Only dives with successful catches were included in this summary.

						mean (s.d.) fish caught per catch location			movement	
prey assemblage	dive type	dive phase	foraging mode	n dives	duration (s) mean (s.d.)	single/ escapees	school edge	school centre	undulate %	rotate %
single	all	complete	sol.	60	57.5 (30.5)	1.6 (1.2)	—	—	90	0
single	all	complete	ass.	5	36.8 (24.8)	1.2 (0.5)	—	—	100	0
single	shallow	shallow	sol.	18	23.2 (15)	1.1 (0.2)	—	—	72	0
single	shallow	shallow	ass.	2	20.5 (13.4)	1	—	—	100	0
single	deep	descent	sol.	39	28.9 (11.4)	0.2 (0.5)	—	—	15.0	0
single	deep	descent	ass.	5	38.6 (25.5)	0	—	—	0	0
single	deep	bottom	sol.	34	19.2 (10.9)	0.7 (1)	—	—	56	0
single	deep	bottom	ass.	0	—	—	—	—	—	—
single	deep	ascent	sol.	40	28.6 (13.5)	1 (1)	—	—	63.0	0
single	deep	ascent	ass.	4	35.3 (5)	1 (0.8)	—	—	75	0
school	all	complete	sol.	29	50.4 (33.4)	3.6 (2.9)	0.6 (0.8)	0.04 (0.2)	93	48
school	all	complete	ass.	19	24.5 (18.7)	3.3 (3.2)	1.1 (1.4)	0.3 (0.5)	89	58
school	shallow	shallow	sol.	13	13.6 (9.3)	1.3 (2.4)	0.5 (0.5)	0.1 (0.3)	92	23
school	shallow	shallow	ass.	11	14.2 (16.4)	0.8 (1.5)	0.9 (0.7)	0.4 (0.5)	82	46
school	deep	descent	sol.	23	24.7 (11)	0.4 (0.9)	0.04 (0.2)	0	22	0
school	deep	descent	ass.	2	21.5 (5)	3.5 (5)	0	0	100	0
school	deep	bottom	sol.	15	16.3 (16.4)	0.6 (1.6)	0.1 (0.3)	0	53	0
school	deep	bottom	ass.	2	17.5 (12)	4.5 (2.1)	0.5 (0.7)	0	100	0
school	deep	ascent	sol.	20	29 (10.8)	3.5 (2.6)	0.5 (0.8)	0	90	50
school	deep	ascent	ass.	5	42.4 (16.7)	7.2 (1.3)	1.8 (2.5)	0.2 (0.4)	100	100

**Figure 2.**
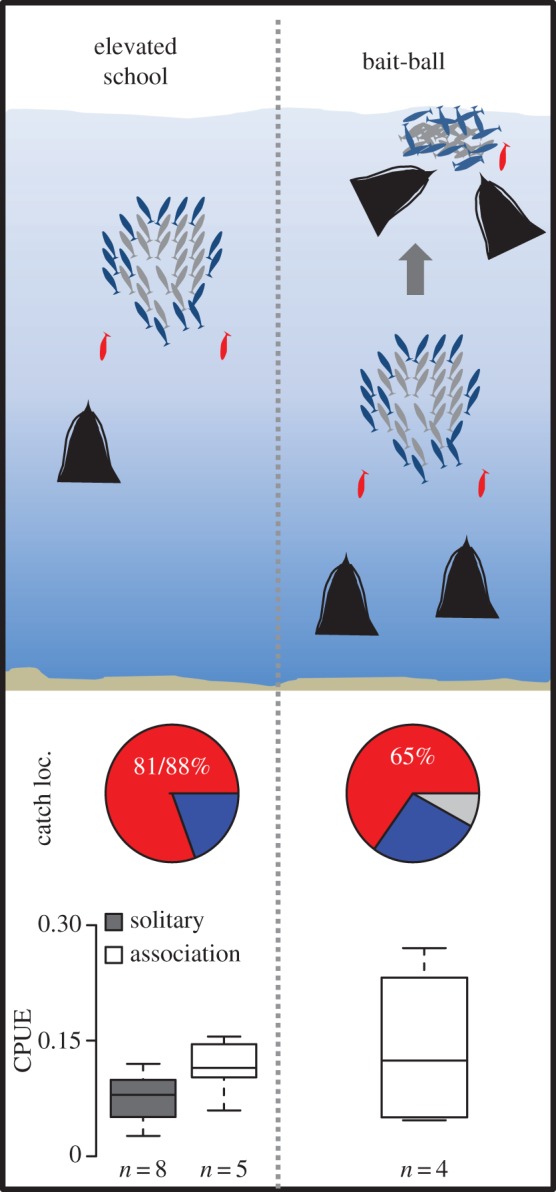
Common prey-pursuit sequences by African penguins on schooling fish (elevated school and bait-ball foraging events) showing proportions of fish caught at different catch locations (catch loc.) in the schools (middle panel): escapees (red), school edge (blue) and school centre (grey). The bottom panel shows box plots comparing foraging efficiency (catch-per-unit-effort) between these foraging events including the influence of foraging mode (solitary versus association) on elevated school events (no solitary events for bait-balls were observed).

**Figure 3.**
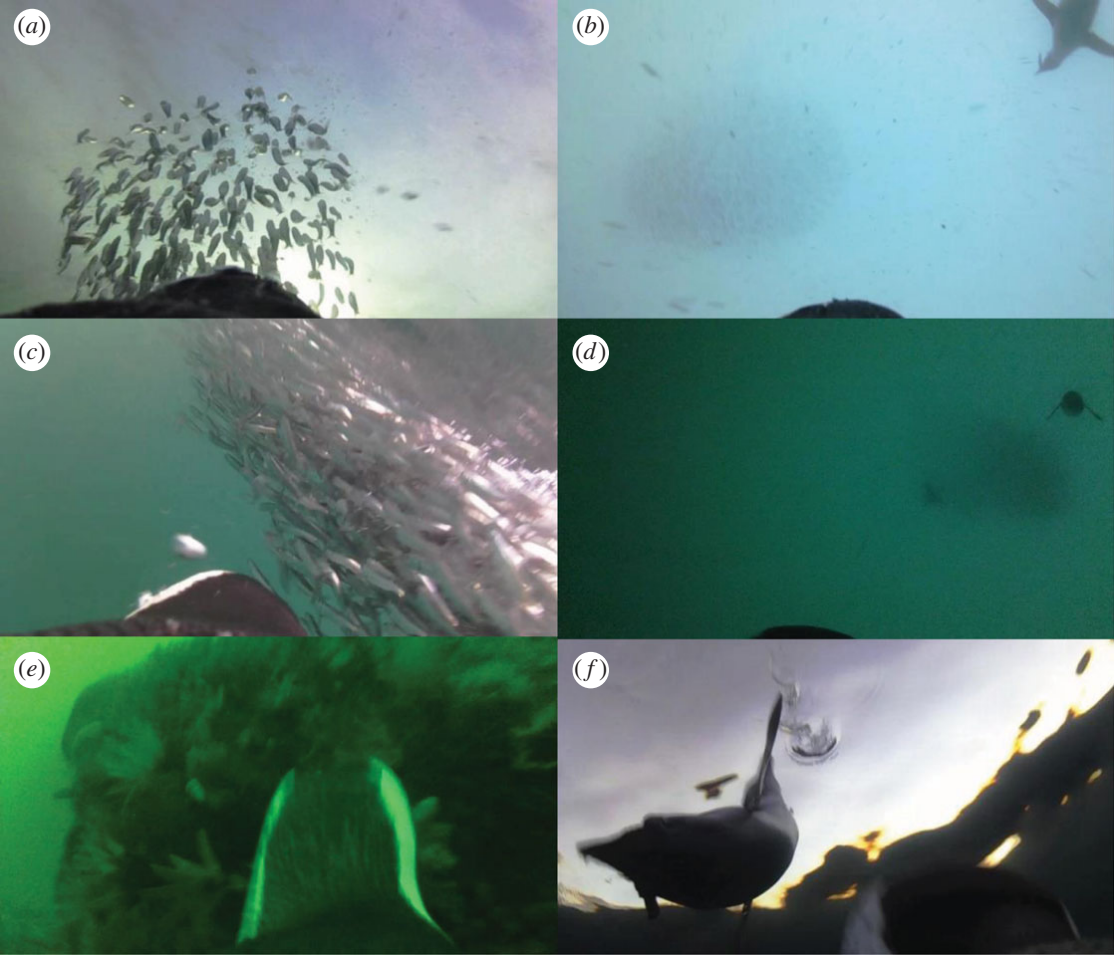
Animal-borne-video-recorder (AVR) images of African penguins: (*a*) pursuit of prey to surface in elevated school event; (*b*) elevated school event with conspecific feeding on escapees; (*c*) bait-ball at surface; (*d*) corralling behaviour of conspecifics; (*e*) investigation of reef substrate, and; (*f*) intraspecific competition with juvenile for single fish prey.

Foraging events involving fish schools included 17 episodes in which schools were located at depths between 11 and 59 m (mean ± s.d.: 34 ± 12 m) and subsequently driven upwards through the water column (figures 2 and 3*a*,*b*); four of these events resulted in bait-balls near the sea surface (figures 2 and 3*c*). The majority of fish taken during these events were escapees (figure 3*b*) and there was an increase in the proportion of fish caught at the edge and in the centre of schools during bait-ball events (figure 2). Corralling behaviour involving conspecifics was recorded in three prey pursuit events (two bait-balls and one elevated school) involving a minimum of 1–3 conspecifics (figure 3*d*, electronic supplementary material, video, Movie S1) and included the largest catch of 19 fish recorded during the study. Multiple dives within the same foraging event occurred on eight occasions, one solitary dive and seven involving conspecifics with this behaviour being more typical of bait-ball events (three of the four) than when fish were not polarized near the surface (range, mean ± s.d. number of dives per event: bait-balls: 3–15, 3 ± 6.4, elevated schools: 1–6, 1 ± 2.2). Less common foraging/searching behaviour included benthic dives in pursuit of small fish schools and the unusual exploration of reef substrate (figure 3*e*). In one instance, direct competition with a juvenile penguin was recorded in pursuit of a single prey item at the surface (figure 3*f*).

### 3.2. Influence of prey aggregation and foraging mode on foraging efficiency

**Figure 4.**
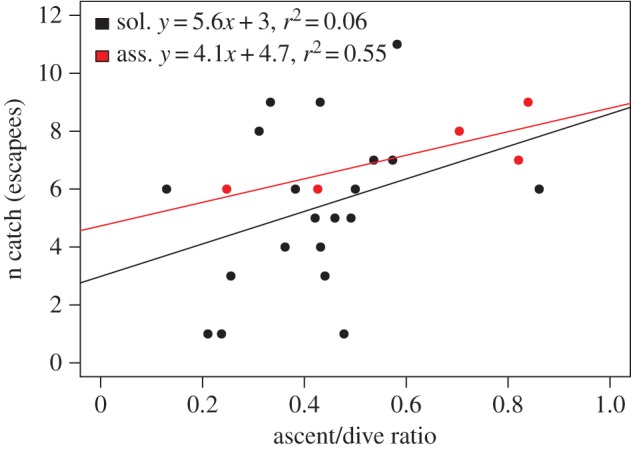
Influence of relative ascent times, i.e. ratio of ascent time to total dive time, to the number of escapee fish caught for solitary African penguins (black) and penguins foraging in groups (red). Linear model fits represent least-square regressions.

Foraging events during which fish schools were encountered were significantly more profitable than those where penguins encountered single prey (median ± inter-quartile range (IQR) catches per foraging event: single prey, 1 ± 1, *N* = 66; school prey, 7 ± 6.5, *N* = 27; Mann–Whitney *U* test, *w* = 1611.5, *p* < 0.001). The most profitable dive phase was ascents involving conspecifics both in terms of the number of fish caught as escapees and, to a lesser extent, fish caught on the edge of schools (table 2). The number of escapees caught was positively related to the proportion of the total dive time made up by the ascent phase, hereafter termed relative ascent time. Least-square regression fits showed that relative ascent time explained a considerably greater amount of variation in the number of escapees caught for birds foraging in groups compared to birds foraging alone (figure 4).

**Figure 5.**
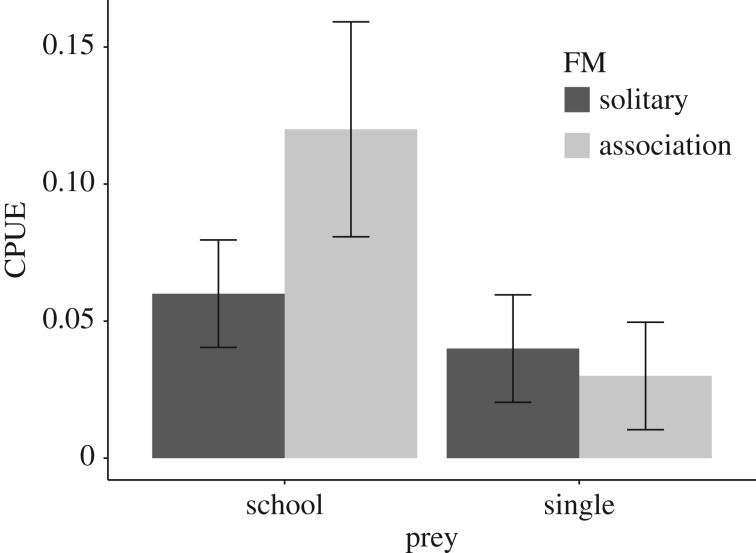
Mean catch-per-unit-effort (CPUE) of African penguins as a function of prey type (single fish versus fish schools) and foraging mode (FM: solitary versus association). Error bars represent the 95% confidence limits.

When controlling for individual effects and foraging mode using the LMM, there was a 1.9× increase in CPUE for penguins catching fish associated with schools as opposed to single fish prey (table 3). Furthermore, there was a significant interaction effect of prey aggregation type and foraging mode on CPUE with foraging efficiency 2.7× greater for birds foraging in groups when catching fish associated with schools compared to catching single fish prey in groups (table 3, figure 5).

**Table 3.** Linear mixed effects model coefficients (*β*) and standard errors (s.e.) for the natural log of catch-per-unit-effort (CPUE) as a function of categorical fixed effects: foraging mode (FM—solitary versus association) and prey aggregation (PA—single versus school) and an interaction effect between these two. Statistics, *t*-values and corresponding *p*-values are given for each variable. *p < 0.05, **p < 0.01, ***p < 0.001.

explanatory variable	*β* (s.e.)	*t*	*p*
intercept	−3.58 (0.13)	−28.67	<0.001***
FM: association	−0.38 (0.26)	−1.46	0.15
PA: school	0.64 (0.23)	2.8	0.007**
CC: assoc. * PA: school	1 (0.39)	2.54	0.013*

For elevated school events, foraging efficiency was improved when penguins foraged in groups although this was not significant (Mann–Whitney *U* test, *w* = 9, *p* = 0.13, figure 2). Bait-ball events produced the most profitable dives, although foraging efficiency was more variable (figure 2).

The estimated number of fish in each school ranged from 26 to 5659 fish (mean ± s.d.: 1079 ± 1415 fish). We found no significant correlation between fish biomass estimates (RFA) for each recording bin with both proxies for the corresponding number of penguins (Spearman's rank correlation, *N* = 10, RFA_max_ versus NCON_max:_: *r* = −0.02, *s* = 168.1, *p* = 0.9; RFA_max_ versus NCON_prop_: *r* = −0.03, *s* = 160, *p* = 0.95; RFA_prop_ versus NCON_max_: *r* = 0.37, *s* = 103.9, *p* = 0.3; RFA_prop_ versus NCON_prop_: *r* = 0.37, *s* = 104, *p* = 0.3).

## 4. Discussion

### 4.1. Group foraging and hunting strategies

To the best of our knowledge this study provides the first quantitative evidence of group foraging benefits in penguins. Although we cannot rule out the possibility that the AVRs had an influence on foraging efficiency, we expect this to be minimal as the cross-sectional area of these devices was 4.4% of that of African penguins, well below the 6.8% threshold previously recommended [31]. Nevertheless, for the purposes of evaluating optimal foraging strategies the comparisons we made involve relative changes in CPUE under the influences of different fixed effects and are therefore expected to be biologically meaningful. We also attempted to control for possible confounding influences of relative prey abundance, albeit rather crude estimates, which had no discernible influence on the propensity for group foraging in African penguins.

The behaviour of foraging African penguins revealed two potential mechanisms supporting group foraging benefits when catching fish associated with schools. Firstly, the most profitable dive phase and hunting technique was the targeting of escapees during the ascent phase of their dives (table 2) with the majority of catches in both elevated school and bait-ball events comprised of these fish (figure 2). Penguins in groups were able to more effectively facilitate the herding of schools upwards through the water column by rotating below and around the sides of the schools (electronic supplementary material, video, Movie S1). Relative ascent times were generally longer for birds foraging in groups. This prolonged the funnelling of schools up through the water column invariably promoting extended periods of escaping behaviour by individual fish and increasing penguin catches (figure 4). Depolarization of schools is advantageous to many fish predators with solitary fish generally being more susceptible to predation (see review [26]). This is possibly due to the moderation of confusion effects associated with anti-predator behaviour by fish in schools [32,33]. African penguins have taken advantage of this vulnerable disposition by inducing high rates of escape as the fish are pushed toward potentially more dangerous zones near the surface.

The second mechanism by which the presence of conspecifics helped improve foraging efficiency was the ability to suspend bait-balls at the surface, a strategy not recorded for solitary foragers during this study. This facilitated up to 15 re-entries into the school extending the time penguins had access to this prey. The formation of bait-balls near the surface is frequently used as a foraging strategy by Delphinids [34]. A positive correlation between predator group size and duration of foraging bouts has been documented for dusky dolphins *Lagenorhynchus obscurus* herding anchovy *Engraulis anchoita* to the surface off Argentina [6]. The limited sample of bait-ball events recorded during our study did not permit an assessment of this nature but the incidence of large numbers of African penguins (mean = 44 birds) corralling bait-balls in Algoa Bay for up to 14 min [22], i.e. 2.8 times the duration of the longest forage event recorded in this study, suggests that group size may be equally important in prolonging foraging bouts for African penguins. This is probably dependent on the prey characteristics, notably species and the size of the fish school.

*In situ* observations of foraging penguins using AVRs or cameras have been recorded for a number of species (mostly generalists), i.e. emperor *Aptenodytes forsteri* [35], gentoo *Pygoscelis papua* [36,37], Adélie *Pygoscelis adeliae* [38–40], chinstrap *Pygoscelis antarcticus* [38], yellow-eyed *Megadyptes antipodes* [41] and little [42] penguins. Group behaviour was recorded for Adélie, chinstrap and little penguins but group foraging was only documented for little penguins where birds rarely caught more than two fish in a dive. Importantly, their foraging efficiency did not improve when foraging with conspecifics [41]. Foraging behaviour complexity is known to vary across the continuum between closely related specialists and generalists [43] which could explain the differences in foraging efficiency between little and African penguins targeting fish in schools. Although African penguins can consume non-pelagic fish prey [44], they predominantly feed on small pelagic fish [16] that are abundant in the nutrient-rich Benguela Upwelling Region. The plumage of African penguins and two of its congeners, Magellanic and Humboldt *Spheniscus humboldti* penguins is likely to have evolved, at least in part, as an adaptation to their piscivorous diet. Countershading in seabirds has been linked to mostly fish-eating species that feed in the mid-water [45,46] and could provide cryptic benefits while pursuing prey [47]. Bold lateral markings in African penguins have been demonstrated to disrupt the schooling behaviour of fish and may help facilitate prey capture [2]. In addition to these morphological adaptations, it is likely that African penguins have evolved specific foraging behaviours, including facilitative group foraging, to maximize prey capture when feeding on schooling fish.

### 4.2. Implications of group activity to African penguin populations

The global population of African penguins decreased by more than 60% since the turn of the century and this trend has been significantly correlated with regional estimates of their prey abundance, mostly anchovy and sardine *Sardinops sagax* [16]. Potential drivers of forage fish population declines around African penguin colonies include resource extraction by the purse-seine fishing industry and the eastward shift in the distribution of anchovy and sardine associated with recent oceanographic changes [48–50]. Inverse density dependence, or Allee effects, can have negative consequences for populations of animals that hunt in groups, especially if this is exacerbated by habitat transformation [51]. The facilitative benefits of group foraging as shown for penguins from Stony Point may be compromised under smaller populations presumably through the diminished probability of locating conspecifics at sea. This situation is likely to be aggravated when shoaling fish are less abundant and less predictable in terms of their distribution. Historically, at a time when populations were significantly larger than today, the majority of African penguins recorded at sea were in groups [23]. The relatively small proportion of group activity recorded during this study, i.e. 35%, may therefore be a reflection of sub-optimal conditions mediated by smaller populations. Furthermore, Allee effects can operate on multiple components of individual fitness [52]. The propensity of African penguins to associate in large groups during surface activity, especially preening, probably incurs additional anti-predator benefits [21]. The degree to which these factors influence demographic parameters [53] has not been assessed and will require detailed comparative analyses of both at-sea behaviour and corresponding survival indices for different population densities. The findings of this research reinforce the need to prevent further population declines of African penguins, which could partly be achieved through the sustainable management of schooling prey resources around penguin breeding colonies.

## Data accessibility

The data, code and video supporting this article can be accessed at the Dryad Digital Repository, (http://dx.doi.org/10.5061/dryad.3j2q3) [54].

## References

[1] Caro T, Beeman K, Stankowich T, Whitehead H 2011 The functional significance of colouration in cetaceans. Evol. Ecol. 25, 1231–1245. (doi:10.1007/s10682-011-9479-5)

[2] Wilson RP, Ryan PG, James A, Wilson MPT 1987 Conspicuous coloration may enhance prey capture in some piscivores. Anim. Behav. 35, 1558–1560. (doi:10.1016/S0003-3472(87)80028-3)

[3] Simmons KEL 1972 Some adaptive features of seabird plumage types. Br. Birds 65, 465–479.

[4] Jurasz CM, Jurasz VP 1979 Feeding modes of the humpback whale*, Megaptera novaeangliae*, in Southeast Alaska. Sci. Rep. Whales Res. Inst. 31, 69–83.

[5] Herbert-Read JE et al. 2016 Proto-cooperation: group hunting sailfish improve hunting success by alternating attacks on grouping prey. Proc. R. Soc. B 283, 20161671 (doi:10.1098/rspb.2016.1671) 10.1098/rspb.2016.1671 PMC5124094 27807269

[6] Wursig B, Wursig M 1980 Behavior and ecology of the dusky dolphin *Lagenorhynchus obscurus* in the south Atlantic. Fish. Bull. 77, 871–890.

[7] Benoit-Bird KJ, Au WWL 2009 Cooperative prey herding by the pelagic dolphin, *Stenella longirostris*. J. Acoust. Soc. Am. 125, 125–137. (doi:10.1121/1.2967480) 19173400 10.1121/1.2967480

[8] Thiebault A, Muller RH., Pistorius PA, Tremblay Y 2014 Local enhancement in a seabird: reaction distances and foraging consequence of predator aggregations. Behav. Ecol. 25, 1302–1310. (doi:10.1093/beheco/aru132)

[9] Götmark F, Winkler DW, Andersson M 1986 Flock-feeding on fish schools increases individual success in gulls. Nature 319, 589–591. (doi:10.1038/319589a0) 3945345 10.1038/319589a0

[10] Thiebault A, Semeria M, Lett C, Tremblay Y, Quinn J 2016 How to capture fish in a school? Effect of successive predator attacks on seabird feeding success. J. Anim. Ecol. 85, 157–167. (doi:10.1111/1365-2656.12455) 26768335 10.1111/1365-2656.12455

[11] Tremblay Y, Cherel Y 1999 Synchronous underwater foraging behavior in penguins. Condor 101, 179–185. (doi:10.2307/1370462)

[12] Takahashi A, Sato K, Nishikawa J, Watanuki Y, Naito Y 2004 Synchronous diving behavior of Adelie penguins. J. Ethol. 22, 5–11. (doi:10.1007/s10164-003-0111-1)

[13] Berlincourt M, Arnould JPY 2014 At-sea associations in foraging little penguins. PLoS ONE 9, e105065 (doi:10.1371/journal.pone.0105065) 25119718 10.1371/journal.pone.0105065 PMC4132066

[14] Allee WC. 1938 Animal aggregations: a study in general sociology. Chicago, IL: University of Chicago Press.

[15] Grunbaum D, Veit R 2017 Black-browed albatrosses foraging on Antarctic krill: density-dependence through local enhancement? Ecology 84, 3265–3275. (doi:10.1890/01-4098)

[16] Crawford RJM et al. 2011 Collapse of South Africa's penguins in the early 21st century. Afr. J. Mar. Sci. 33, 139–156. (doi:10.2989/1814232X.2011.572377)

[17] Wilson RP 1985 Seasonality in diet and breeding success of the jackass penguin *Spheniscus demersus*. J. Ornithol. 126, 53–62. (doi:10.1007/BF01640442)

[18] Wilson RP 1985 The jackass penguin (*Spheniscus demersus*) as a pelagic predator. Mar. Ecol. 25, 219–227. (doi:10.3354/meps025219)

[19] Petersen SL, Ryan PG, Gremillet D 2006 Is food availability limiting African penguins *Spheniscus demersus* at Boulders? A comparison of foraging effort at mainland and island colonies. Ibis 148, 14–26. (doi:10.1111/j.1474-919X.2006.00459.x)

[20] Pichegru L, Cook T, Handley J, Voogt N, Watermeyer J, Nupen L, McQuaid C 2013 Sex-specific foraging behaviour and a field sexing technique for endangered African penguins. Endanger. Species Res. 19, 255–264. (doi:10.3354/esr00477)

[21] Wilson RP, Wilson M-PT, McQuaid L 1986 Group size in foraging African penguins (*Spheniscus demersus*). Ethology 72, 338–341. (doi:10.1111/j.1439-0310.1986.tb00634.x)

[22] Ryan P, Edwards L, Pichegru L 2012 African penguins *Spheniscus demersus*, bait balls and the Allee effect. Ardea 100, 89–94. (doi:10.5253/078.100.0113)

[23] Siegfried WR, Frost PGH, Kinahan JB, Cooper J 1975 Social behaviour of jackass penguins at sea. Zool. Africana 10, 87–100. (doi:10.1080/00445096.1975.11447495)

[24] Wilson RP, Wilson MT. 1990 Foraging ecology of breeding *Spheniscus* penguins. In Penguin biology (eds Davis LS, Darby JT), pp. 181–206. San Diego, CA: Academic Press Inc..

[25] Simeone A, Wilson RP 2003 In-depth studies of Magellanic penguin (*Spheniscus magellanicus*) foraging: can we estimate prey consumption by perturbations in the dive profile? Mar. Biol. 143, 825–831. (doi:10.1007/s00227-003-1114-8)

[26] Pitcher TJ, Parrish JK. 1993 Functions of shoaling behaviour in teleosts. In Behaviour of teleost fishes (ed. TJ Pitcher), p. 715 London, UK: Chapman & Hall.

[27] Ryan P, Petersen S, Simeone A, Gremillet D 2007 Diving behaviour of African penguins: do they differ from other *Spheniscus* penguins? Afr. J. Mar. Sci. 29, 153–160. (doi:10.2989/AJMS.2007.29.2.1.184)

[28] R Core Team. 2015 R: a language and environment for statistical computing. See http://www.R-project.org/.

[29] Pinheiro J, Bates D, DebRoy S, Sarkar D, R Core Team. 2015 {nlme}: Linear and nonlinear mixed effects models. See https://cran.r-project.org/web/packages/nlme/index.html.

[30] Carroll G, Cox M, Harcourt R, Pitcher BJ, Slip D, Jonsen I, Boogert N 2017 Hierarchical influences of prey distribution on patterns of prey capture by a marine predator. Funct. Ecol. 31, 1750–1760. (doi:10.1111/1365-2435.12873)

[31] Wilson RP, Grant WS, Duffy DC 1986 Recording devices on free-ranging marine animals: does measurement affect foraging performance? Ecology 67, 1091–1093. (doi:10.2307/1939832)

[32] Norris KS, Schilt CR 1988 Cooperative societies in three-dimensional space: on the origins of aggregations, flocks, and schools, with special reference to dolphins and fish. Ethol. Sociobiol. 9, 149–179. (doi:10.1016/0162-3095(88)90019-2)

[33] Milinski M 1979 Can an experienced predator overcome the confusion of swarming prey more easily? Anim. Behav. 27, 1122–1126. (doi:10.1016/0003-3472(79)90060-5)

[34] Wiirsig B. 1986 Delphinid foraging strategies. In Dolphin cognition and behavior: a comparative approach (eds Schusterman R, Thomas J, Wood F), pp. 347–359. New York, NY: Lawrence Erlbaum Associates, Inc.

[35] Ponganis PJ, Van Dam RP, Marshall G, Knower T, Levenson DH 2000 Sub-ice foraging behavior of emperor penguins. J. Exp. Biol. 203, 3275–3278. 11023847 10.1242/jeb.203.21.3275

[36] Takahashi A, Kokubun N, Mori Y, Shin HC 2008 Krill-feeding behaviour of gentoo penguins as shown by animal-borne camera loggers. Polar Biol. 31, 1291–1294. (doi:10.1007/s00300-008-0502-4)

[37] Handley JM, Pistorius P 2016 Kleptoparasitism in foraging gentoo penguins *Pygoscelis papua*. Polar Biol. 39, 391–395. (doi:10.1007/s00300-015-1772-2)

[38] Takahashi A, Sato K, Naito Y, Dunn MJ, Trathan PN, Croxall JP 2004 Penguin-mounted cameras glimpse underwater group behaviour. R. Soc. Biol. Lett. 271, 281–282. (doi:10.1098/rsbl.2004.0182) 10.1098/rsbl.2004.0182 PMC1810073 15503994

[39] Watanabe YY, Takahashi A 2013 Linking animal-borne video to accelerometers reveals prey capture variability. Proc. Natl Acad. Sci. USA 110, 2199–2204. (doi:10.1073/pnas.1216244110) 23341596 10.1073/pnas.1216244110 PMC3568313

[40] Thiebot JB, Ito K, Raclot T, Poupart T, Kato A, Ropert-Coudert Y, Takahashi A 2016 On the significance of Antarctic jellyfish as food for Adelie penguins, as revealed by video loggers. Mar. Biol. 163, 1–8. (doi:10.1007/s00227-016-2890-2)

[41] Mattern T, McPherson MD, Ellenberg U, van Heezik Y, Seddon PJ. 2017 High definition video loggers provide new insights into behaviour, physiology, and the oceanic habitat of marine top predators. PeerJ Prepr. 5, e2765v2 (doi:10.7287/peerj.preprints.2765v2) 10.7717/peerj.5459 PMC6151119 30258706

[42] Sutton GJ, Hoskins AJ, Arnould JPY, Hazen EL 2015 Benefits of group foraging depend on prey type in a small marine predator, the little penguin. PLoS ONE 10, e0144297 (doi:10.1371/journal.pone.0144297) 26674073 10.1371/journal.pone.0144297 PMC4682954

[43] Drummond H 1983 Aquatic foraging in garter snakes: a comparison of specialists and generalists. Behaviour 86, 1–30. (doi:10.1163/156853983X00543)

[44] Connan M, Hofmeyr GJG, Pistorius PA, Ropert-Coudert Y. 2016 Reappraisal of the trophic ecology of one of the world's most threatened spheniscids, the African penguin. PLoS ONE 11, e0159402 (doi:10.1371/journal.pone.0159402) 27434061 10.1371/journal.pone.0159402 PMC4951110

[45] Bretagnolle V 1993 Adaptive significance of seabird colouration: the case of Procellariiforms. Am. Nat. 142, 141–173. (doi:10.1086/285532) 19425973 10.1086/285532

[46] Cairns DK 1986 Plumage colour in pursuit-diving seabirds: why do penguins wear tuxedos? Bird Behav. 6, 28–65. (doi:10.3727/015613886792195225)

[47] Ruxton GD, Speed MP, Kelly DJ 2004 What, if anything, is the adaptive function of countershading? Anim. Behav. 68, 445–451. (doi:10.1016/j.anbehav.2003.12.009)

[48] Roy C, van der Lingen CD, Coetzee JC, Lutjeharms JRE 2007 Abrupt environmental shift associated with changes in the distribution of Cape anchovy *Engraulis encrasicolus* spawners in the southern Benguela. Afr. J. Mar. Sci. 29, 309–319. (doi:10.2989/AJMS.2007.29.3.1.331)

[49] Coetzee JC, van der Lingen CD, Hutchings L, Fairweather TP 2008 Has the fishery contributed to a major shift in the distribution of South African sardine? ICES J. Mar. Sci. 65, 1676–1688. (doi:10.1093/icesjms/fsn184)

[50] Crawford RJM, Makhado AB, Whittington PA, Randall RM, Oosthuizen WH, Waller LJ 2015 A changing distribution of seabirds in South Africa—the possible impact of climate and its consequences. Front. Ecol. Evol. 3, 1–11. (doi:10.3389/fevo.2015.00010)

[51] Courchamp F, Clutton Brock T, Grenfell B 2000 Multipack dynamics and the Allee effect in the African wild dog, *Lycaon pictus*. Anim. Conserv. 3, 277–285. (doi:10.1111/j.1469-1795.2000.tb00113.x)

[52] Berec L, Angulo E, Courchamp F 2007 Multiple Allee effects and population management. Trends Ecol. Evol. 22, 185–191. (doi:10.1016/j.tree.2006.12.002) 17175060 10.1016/j.tree.2006.12.002

[53] Kramer AM, Dennis B, Liebhold AM, Drake JM 2009 The evidence for Allee effects. Popul. Ecol. 51, 341–354. (doi:10.1007/s10144-009-0152-6)

[54] McInnes AM, McGeorge C, Ginsberg S, Pichegru L, Pistorius PA 2017 Data from: Group foraging increases foraging efficiency in a piscivorous diver, the African penguin Dryad Digital Repository. (http://dx.doi.org/10.5061/dryad.3j2q3) 10.1098/rsos.170918 PMC5627125 28989785

